# Use of DNA–Damaging Agents and RNA Pooling to Assess Expression Profiles Associated with *BRCA1* and *BRCA2* Mutation Status in Familial Breast Cancer Patients

**DOI:** 10.1371/journal.pgen.1000850

**Published:** 2010-02-19

**Authors:** Logan C. Walker, Bryony A. Thompson, Nic Waddell, kConFab Investigators, Sean M. Grimmond, Amanda B. Spurdle

**Affiliations:** 1 Genetics and Population Health Division, Queensland Institute of Medical Research, Brisbane, Australia; 2 Peter MacCallum Cancer Centre, Melbourne, Australia; 3 Genomics and Computational Biology Division, Institute for Molecular Biosciences, University of Queensland, Brisbane, Australia; Harvard Medical School and Brigham and Women's Hospital, United States of America

## Abstract

A large number of rare sequence variants of unknown clinical significance have been identified in the breast cancer susceptibility genes, *BRCA1* and *BRCA2.* Laboratory-based methods that can distinguish between carriers of pathogenic mutations and non-carriers are likely to have utility for the classification of these sequence variants. To identify predictors of pathogenic mutation status in familial breast cancer patients, we explored the use of gene expression arrays to assess the effect of two DNA–damaging agents (irradiation and mitomycin C) on cellular response in relation to *BRCA1* and *BRCA2* mutation status. A range of regimes was used to treat 27 lymphoblastoid cell-lines (LCLs) derived from affected women in high-risk breast cancer families (nine *BRCA1*, nine *BRCA2*, and nine non-*BRCA1/2* or BRCAX individuals) and nine LCLs from healthy individuals. Using an RNA–pooling strategy, we found that treating LCLs with 1.2 µM mitomycin C and measuring the gene expression profiles 1 hour post-treatment had the greatest potential to discriminate *BRCA1*, *BRCA2*, and BRCAX mutation status. A classifier was built using the expression profile of nine QRT–PCR validated genes that were associated with *BRCA1*, *BRCA2*, and BRCAX status in RNA pools. These nine genes could distinguish *BRCA1* from *BRCA2* carriers with 83% accuracy in individual samples, but three-way analysis for *BRCA1*, *BRCA2*, and BRCAX had a maximum of 59% prediction accuracy. Our results suggest that, compared to *BRCA1* and *BRCA2* mutation carriers, non-*BRCA1/2* (BRCAX) individuals are genetically heterogeneous. This study also demonstrates the effectiveness of RNA pools to compare the expression profiles of cell-lines from *BRCA1*, *BRCA2*, and BRCAX cases after treatment with irradiation and mitomycin C as a method to prioritize treatment regimes for detailed downstream expression analysis.

## Author Summary

A large number of rare sequence variants of unknown clinical significance have been identified in the breast cancer susceptibility genes, *BRCA1* and *BRCA2*. Laboratory methods to identify which of these variants are mutations would have utility for counseling and clinical decision making when identified in patients with a family history of breast cancer. We used DNA–damaging agents to disturb gene expression profiles of cell-lines derived from blood of patients, and we compared patterns from women with *BRCA1* and *BRCA2* mutations to women familial breast cancer families without such mutations. Using a pooling strategy, which allowed us to compare several treatments at one time, we identified which treatment caused the greatest difference in gene-expression changes between patient groups and used this treatment method for further study. We were able to accurately classify *BRCA1* and *BRCA2* samples, and our results supported other reported findings that suggested familial breast cancer patients without *BRCA1/2* mutations are genetically heterogeneous. We demonstrate a useful strategy to identify treatments that induce gene expression differences associated with *BRCA1/2* mutation status. This strategy may aid the development of a molecular-based tool to screen individuals from multi-case breast cancer families for the presence of pathogenic mutations.

## Introduction

Rare sequence variants in *BRCA1* and *BRCA2* that are not predicted to lead to obvious or easily detectable molecular aberrations, such as protein truncation or RNA splicing defects, are currently difficult to classify clinically as pathogenic or neutral. These variants attribute to approximately 10% of clinical test results, and create a significant challenge for counseling and clinical decision making when identified in patients with a strong family history of breast cancer. Laboratory based methods that can distinguish between carriers of known pathogenic mutations and non-carriers are likely to have utility for the classification of sequence variants of unknown clinical significance.

Expression profiling has been used successfully to characterize molecular subtypes in breast cancer whether based on gene expression patterns in primary tumor cells [1]–[3], metastatic cells [4], or stroma-derived cells [5]. Distinctive patterns of global gene expression have also been shown between breast tumors with *BRCA1* mutations and breast tumors with *BRCA2* mutations [6]. More recently, evidence has been presented from several studies to suggest that heterozygous carriers of *BRCA1* and *BRCA2* mutations, and breast cancer patients without such alterations may be distinguished based on mRNA profiling of fibroblasts and lymphoblastoid cell-lines (LCLs) [7]–[9]. In one study, short-term breast fibroblast cell-lines were established from nine individuals with a *BRCA1* germ-line mutation, and five healthy control individuals with no personal or family history of breast cancer [7]. Class prediction analysis using expression data from irradiated fibroblast cultures showed that *BRCA1* carriers could be distinguished from controls with 85% accuracy [7]. A similar study used short-term fibroblast cultures from skin biopsies from 10 *BRCA1* and 10 *BRCA2* mutation carriers and 10 individuals who had previously had breast cancer but were unlikely to contain *BRCA1/2* mutations [8]. Class prediction analysis using expression data from irradiated fibroblast cultures showed that *BRCA1* and *BRCA2* samples could be classified with 95% accuracy, and *BRCA1/2* carriers could be distinguished from noncarriers with 90% to 100% accuracy [8].

In contrast to short-term fibroblast cell-lines, lymphoblastoid cell-lines (LCLs) are a minimally invasive source of germline material that can be maintained as long term culture, and which have proven to be a valuable model system for studying gene expression signatures in relation to genetic variation and external stimulants [10]–[13]. A recent study from our laboratory utilizing this model system suggested that post-irradiation (IR) gene expression data from LCLs derived from blood of patients with sequence alterations in *BRCA1* and *BRCA2*, and from familial breast cancer patients without such alterations (BRCAX) has potential to predict *BRCA1*, *BRCA2* and BRCAX mutation status with up to 62% accuracy [9]. In view of improving prediction accuracy, especially between *BRCA1* and BRCA2, we used expression arrays to assess the effect of the DNA damaging agents, IR and mitomycin C (MMC), at different time points, on cellular response in relation to mutation status. To facilitate analysis of the large number of treated LCLs, an RNA pooling strategy was implemented to reduce the number of microarray experiments by three-fold. Previous studies have used RNA pooling as a strategy to reduce the effects of biological variation in order to help identify key features that differ between biological class [14],[15]. We have therefore explored a similar approach in this study using patient derived LCLs as well as prior knowledge that LCL expression profiles are influenced by both genotype and exogenous factors. This strategy was shown to be effective in identifying genes dysregulated in response to DNA damaging agents. This study also demonstrated the effectiveness of RNA pools to compare the effect of various IR and MMC treatment regimes on the mRNA expression profiles of LCLs derived from *BRCA1*, *BRCA2* and BRCAX cases for downstream detailed analysis of individual samples.

## Results

### Effect of IR and MMC on global gene expression

**Figure 1:**
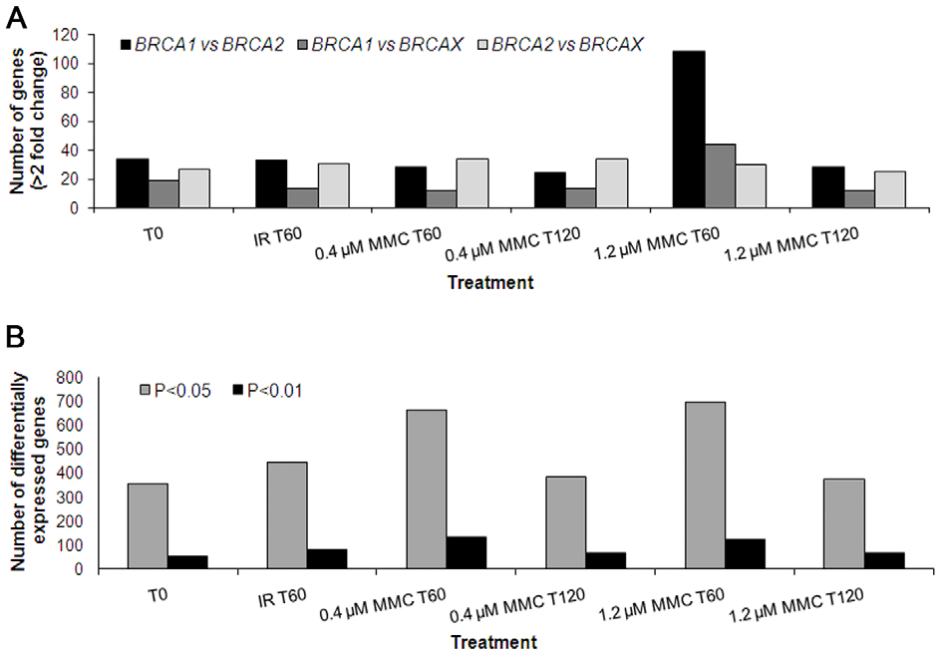
Number of genes differentially expressed among *BRCA1*, *BRCA2*, and BRCAX. Differential expression was determined by (A) fold-change (geometric mean of the expression ratios >2), and (B) statistical correlation using the F-test and alpha levels 0.05 and 0.01.

To identify which treatment caused the greatest amount of change in gene expression levels, we first determined the number of genes that showed differential expression between pools for each treatment, particularly for *BRCA1 versus BRCA2* and *BRCA1 versus* BRCAX (Figure 1). Using fold-change as a measure of differential gene expression revealed that the number of genes differentially expressed (>2-fold) between *BRCA1*, *BRCA2* and BRCAX pools after IR was similar to that shown by the untreated controls (Figure 1A). However, significant differences in the expression of genes acting in the IR-induced ATM signaling pathway was observed between irradiated LCL pools compared to untreated pools (Figure S1), confirming inducement of an expression phenotype by IR. Of the four MMC treatments, the number of genes differentially expressed between pools was greatest when LCLs were treated with 1.2 µM MMC and the RNA isolated 1 hour post-treatment (Figure 1A). There is currently no canonical or consensus pathway based on MMC activity. It was therefore not possible to confirm the effects of this treatment by assessing expression phenotypes.

Identifying which treatment produced expression change in the greatest number of genes was also carried out by performing an F-test separately for each gene and determining the number of genes differentially expressed with significance levels set at 0.05 and 0.01 (Figure 1B). LCLs treated with MMC showed the greatest expression change after 1 hour incubation, with a slightly bigger effect associated with 1.2 µM MMC *versus* 0.4 µM MMC, thus suggesting that MMC has a greater perturbation effect after the shorter incubation period (Figure 1B). A similar trend in the number of genes differentially expressed between classes was observed when the significance level was set at 0.001 (Data not shown). Overall, these results indicated that, of the treatments used, 1.2 µM MMC(T_60_) was most likely to induce gene expression profiles that differ significantly between *BRCA1*, *BRCA2* and BRCAX LCLs.

### Identification of MMC responsive genes that discriminate *BRCA1*, *BRCA2*, and BRCAX mutation type

**Figure 2:**
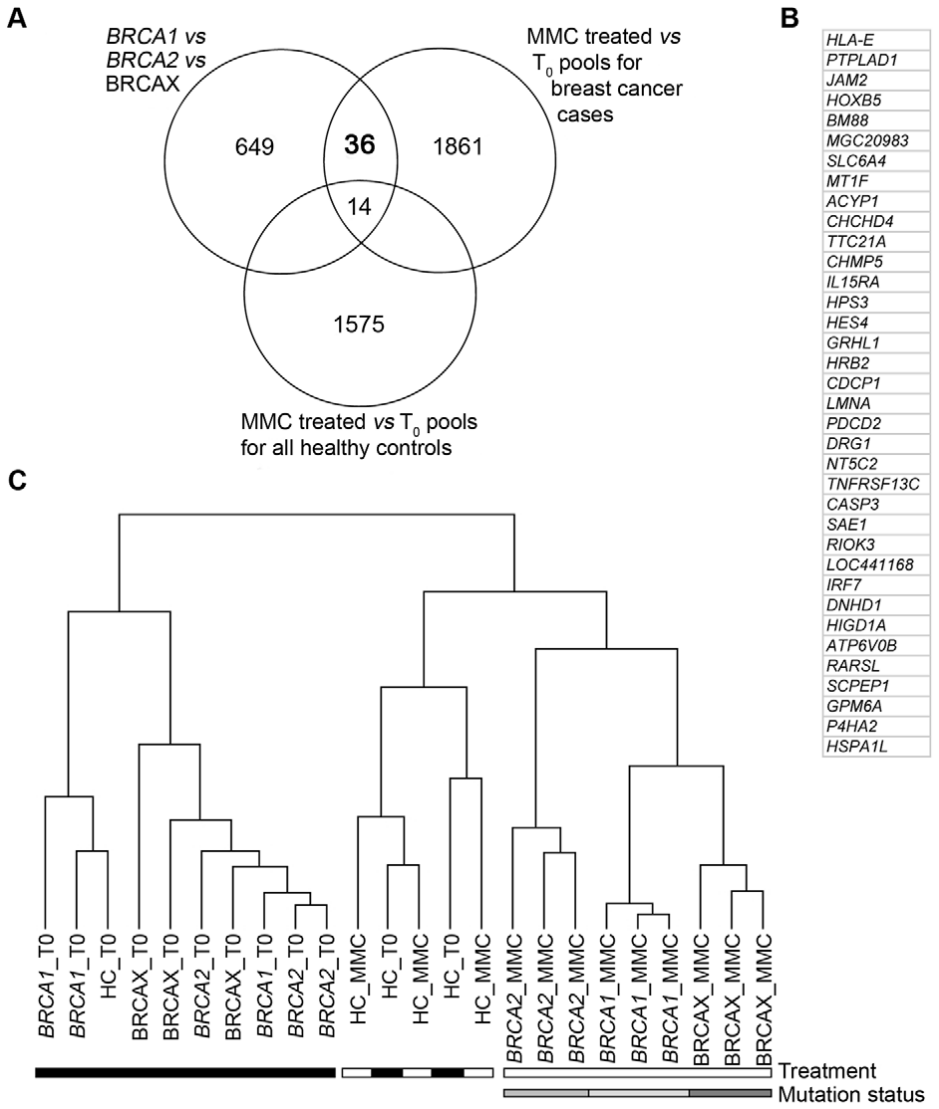
Classifying *BRCA1*, *BRCA2*, and BRCAX subtype by MMC response genes. (A) Venn diagram illustrating the number of genes identified from three analyses: 1) 3-way comparison of *BRCA1*, *BRCA2* and BRCAX pools (F-test, P<0.05); 2) Pairwise comparison of 1.2 µM MMC-T_60_ treated and non-treated *BRCA1/2*/X pools (<10% false discovery rate; 90% confidence level); and 3) 2-way comparison of 1.2 µM MMC-T_60_ treated and non-treated healthy control pools (T-test, P<0.05). The extent of overlap between gene lists is shown. (B) List of 36 genes that are differentially expressed between *BRCA1*, *BRCA2*, and BRCAX, and are MMC responsive in affected carrier pools but not in healthy controls. (C) Supervised hierarchical clustering of treated (1.2 µM MMC-T_60_) sample pools using the 36-gene list.

To identify genes that would discriminate pools based on mutation status, three comparative analyses were performed to achieve three objectives. The first objective was to identify genes that were differentially expressed between *BRCA1*, *BRCA2* and BRCAX pools treated with 1.2 µM MMC(T_60_). This analysis identified 699 genes that are able to discriminate pools based on mutation status (Figure 2A, Table S1). The second objective was to identify genes that were differentially expressed between treated 1.2 µM MMC(T_60_) and non-treated *BRCA1*, *BRCA2* and BRCAX pools. The 1911 genes identified from this analysis were then characterized as MMC responsive (Figure 2A, Table S1). Combining these two analyses revealed 50 genes that classified pools based on mutation status and that are also MMC responsive (Figure 2A). The third objective was to identify genes that were differentially expressed between treated (1.2 µM MMC(T_60_)) and non-treated healthy control pools. This analysis was important to identify genes that are MMC responsive in healthy controls and therefore not specific for mutation status in *BRCA1*, *BRCA2* and BRCAX pools (Figure 2A, Table S1). By combining the results of these three analyses, 36 genes were identified that are differentially expressed between *BRCA1*, *BRCA2* and BRCAX pools, and are also MMC responsive in affected carrier pools but not in healthy controls (Figure 2A and 2B). As expected, supervised hierarchical clustering of 1.2 µM MMC(T_60_) treated and non-treated pools using the 36-gene list demonstrates a separation of treated pools based on mutation type, but no separation by mutation type was observed in untreated pools (Figure 2C). Likewise, there was no discrimination of treated and untreated healthy control pools (Figure 2C).

QRT-PCR was carried out to validate the expression levels of the 36 MMC responsive genes in the *BRCA1*, *BRCA2*, and BRCAX derived RNA pools. Despite relatively small fold-changes detected in pools for each of the 36 genes between the three mutation groups, 15 genes were validated by QRT-PCR (Table 1); three times more than that expected by chance.

**Table 1:** Fifteen QRT–PCR validated genes shown to be differentially expressed among *BRCA1*, *BRCA2*, and BRCAX pools, and MMC responsive in affected carrier pools but not in healthy controls.

Gene Symbol	*BRCA1*/*BRCA2* ^a^		*BRCA1*/BRCAX^a^		*BRCA2*/BRCAX^a^		Pearson's correlation^b^
	Microarray	QRT-PCR	Microarray	QRT-PCR	Microarray	QRT-PCR	
*SLC6A4*	**1.08**	**1.23**	**0.66**	**0.52**	**0.61**	**0.43**	**0.95**
*FAM26F*	**0.39**	**0.60**	**0.63**	**0.94**	**1.63**	**1.55**	**0.83**
*CCDC151*	**0.51**	**0.51**	**0.48**	**0.50**	**0.95**	**0.99**	**0.80**
*RARSL*	**1.14**	**1.32**	**1.00**	**1.18**	**0.88**	**0.90**	**0.75**
*P4HA2*	**0.61**	**0.49**	**0.97**	**0.65**	**1.60**	**1.33**	**0.70**
*LMNA*	**1.47**	**1.27**	**1.82**	**1.71**	**1.24**	**1.35**	**0.68**
*CASP3*	**1.56**	**1.28**	**1.30**	**1.03**	**0.83**	**0.81**	**0.67**
*GPM6A*	**0.91**	**0.34**	**0.94**	**0.75**	**1.04**	**2.18**	**0.61**
*CDCP1*	**0.98**	**0.81**	**1.18**	**2.14**	**1.21**	**2.63**	**0.61**
*CEND1*	1.10	1.20	0.86	0.80	0.79	0.67	0.48
*TNFRSF13C*	0.59	0.95	0.66	0.98	1.13	1.04	0.47
*HES4*	0.38	0.58	0.69	0.69	1.82	1.19	0.47
*CHCHD4*	1.01	1.07	0.75	0.87	0.74	0.81	0.42
*HSPA1L*	0.87	0.83	0.83	0.79	0.96	0.95	0.35
*GRHL1*	0.96	0.81	0.83	0.70	0.87	0.86	0.34

**a** Ratio of the average expression level.

**b** Correlation between microarray and QRT–PCR expression data from nine RNA pools. Nine genes with a Pearson's correlation greater than 0.6 are shown in bold.

Of these 15 genes, nine also showed high correlation (r>0.6) in expression level between microarray and the QRT-PCR value of the same RNA pools (Table 1). These nine MMC responsive genes were therefore selected for class prediction tests.

### Comparison of RNA pools and virtual pools

**Figure 3:**
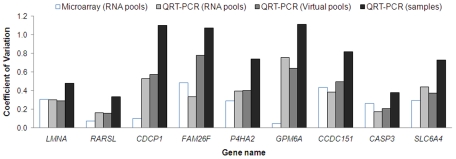
The coefficient of variation (i.e. standard deviation divided by the mean) of the expression values for the nine MMC responsive genes. For each gene, microarray and/or QRT–PCR derived data are compared across RNA pools, virtual pools and individual samples.

To explore potential technical variation associated with generating RNA pools, we compared expression levels of the nine MMC responsive genes, measured by microarray and QRT-PCR analysis in the nine RNA pools, and by QRT-PCR in the 27 individual LCL samples. Virtual pools were also generated by taking the average of QRT-PCR expression values from the individual samples used in the pools. Figure 3 shows that the coefficient of variation (CV) differed between the nine genes regardless of the experiment strategy. The least amount of variation from measured gene expression tended to be observed after microarray analysis of RNA pools with the CV ranging from 0.05 to 0.49 for the nine validated genes (Figure 3). In contrast, the greatest amount of variation from measured gene expression tended to be observed after QRT-PCR analysis of individual RNA samples with the CV of the same genes ranging from 0.33 to 1.11 (Figure 3). Similar gene expression variation was observed between RNA pools (CV ranged from 0.16 to 0.76) and virtual pools (CV ranged from 0.16 to 0.78), with the exception of *FAM26F* (Figure 3). Moreover, the correlation of expression data between the RNA pools and virtual pools was greater than 0.7 for seven of the nine genes analyzed (Table S2). These results suggest that although pooling reduces measured variation in expression levels, this reduction is most likely the result of a biological averaging effect and not technical issues relating to the different steps involved in the microarray experiment.

### Class prediction of *BRCA1*, *BRCA2*, and BRCAX mutation status using nine MMC responsive genes

We utilized five different prediction methods (Diagonal Linear Discriminant Analysis, 1-Nearest Neighbour and Nearest Centroid classification, Support Vector Machines, and Compound Covariate Predictor) to determine the accuracy of using the nine MMC responsive genes to predict the three biological classes (*BRCA1* truncation mutation, *BRCA2* truncation mutation, and BRCAX) by means of a three-way comparison (Details shown in Table S3, S4). If the nine genes selected for classification are related to MMC response pathways we would predict better predictions for MMC treated groups compared to non-treated and IR treated group. Interestingly, *BRCA1*, *BRCA2* and BRCAX pools were poorly classified from the IR(T_60_) (44%–78%) and T_0_ (33%–56%) treatments groups compared to the four MMC treated groups (67%–100%) using microarray data from the nine MMC responsive genes (Table S5). Results from the class prediction analysis of 1.2 µM MMC(T_60_) treated pools and individual samples are shown in Table 2. Not surprisingly, the highest accuracy (67%–100%) for predicting *BRCA1*, *BRCA2* and BRCAX mutation status of pools was achieved using microarray data (Table 2). By comparison, performing the same analysis on *BRCA1*, *BRCA2* and BRCAX pools using QRT-PCR derived expression data achieved an accuracy of 56%-78% in predicting mutation type for each of the pooled RNAs (Table 2).

**Table 2:** Accuracy of class prediction based on the expression profile of nine MMC responsive genes.

Class	Expression data source^a^	Mean percent of correct classification				
		DLDA	1-NN	NC	SVM	CCP
***BRCA1*** **vs** ***BRCA2*** **vs** **BRCAX**	Pools (Microarray)	100%	67%	89%	–	–
	Pools (QRT-PCR)	56%	67%	78%	–	–
	Virtual Pools (QRT-PCR)	78%	89%	67%	–	–
	Samples (QRT-PCR)	48%	52%	59%	–	–
***BRCA1*** **vs** ***BRCA2***	Pools (Microarray)	83%	100%	100%	100%	100%
	Pools (QRT-PCR)	100%	100%	100%	83%	100%
	Virtual Pools (QRT-PCR)	100%	100%	100%	100%	100%
	Samples (QRT-PCR)	83%	83%	83%	83%	83%
***BRCA1*** **vs** **BRCAX**	Pools (Microarray)	100%	100%	100%	100%	100%
	Pools (QRT-PCR)	67%	83%	67%	83%	67%
	Virtual Pools (QRT-PCR)	83%	100%	83%	100%	83%
	Samples (QRT-PCR)	56%	56%	67%	56%	56%
***BRCA2*** **vs** **BRCAX**	Pools (Microarray)	100%	50%	83%	83%	100%
	Pools (QRT-PCR)	67%	67%	83%	50%	67%
	Virtual Pools (QRT-PCR)	33%	83%	67%	67%	83%
	Samples (QRT-PCR)	44%	72%	72%	61%	72%

**a** Pools, n = 9; Samples, n = 27. Abbreviations: CCP, Compound covariate predictor; DLDA, Diagonal Linear Discriminant Analysis; NC, Nearest Centroid; 1-NN, Nearest Neighbour; SVM, support vector machine.

Prediction analysis with QRT-PCR data from the 27 individual LCL samples used to derive the nine *BRCA1*, *BRCA2*, and BRCAX associated pools correctly classified the individual samples with up to 59% accuracy using the NC model (Table 2). Similar to the results shown using QRT-PCR data from the RNA pools, classification of the virtual pools was typically lower than that seen with the microarray data but higher than that achieved when analyzing the individual samples (Table 2). In addition to the three-way comparison, we also performed a series of two-way comparisons to explore the accuracy of the nine MMC responsive genes to classify both pools and individual samples (Details shown in Table S6, S7, S8, S9, S10, S11). Notably, these genes were sufficient to classify *BRCA1 versus BRCA2* pools with 100% accuracy, with a slightly reduced prediction accuracy of 83% within the individual samples for all models (Table 2). Classification was lowest when comparing BRCAX and *BRCA1* samples (56%–67%), or BRCAX and *BRCA2* samples (44%–72%) (Table 2). Although the DLDA classifier performed well using microarray derived expression values from *BRCA1*, *BRCA2* and BRCAX pools, the model performed relatively poorly when classifying individual samples with greater than 50% misclassification in some analyses (Table 2). The best performing classifier of individual samples was the Nearest Centroid model which gave the highest prediction accuracy (59%) for *BRCA1*, *BRCA2* and BRCAX mutation type. Furthermore, this model successfully classified the majority of individual samples from two-way comparisons of *BRCA1 versus BRCA2* (83%), *BRCA1 versus* BRCAX (67%), and *BRCA2 versus* BRCAX (72%).

## Discussion

We have recently reported a study using expression profiling of IR treated LCLs to predict the mutations status of *BRCA1* and *BRCA2* with the ultimate aim of predicting the significance of unclassified variants of *BRCA1* and *BRCA2* [9]. Using similar rationale, the present study explores the use of treatment regimes that employ the DNA damaging agents, IR and MMC, with the aim to increase the prediction accuracy from that reported by Waddell et al, especially between *BRCA1* and *BRCA2* [9]. Furthermore, this study demonstrates the use of RNA pools to compare the effect of five different IR or MMC treatment regimes on the expression profiles of LCLs derived from *BRCA1*, *BRCA2* and BRCAX cases.

Our results from analysis of RNA pools suggested that treating LCLs with 1.2 µM MMC and measuring the gene expression profiles 60 minutes post-treatment had the greatest potential to discriminate *BRCA1*, *BRCA2* and BRCAX mutation status. We subsequently built a classifier using the expression of nine genes that were responsive to the 1.2 µM MMC(T_60_) treatment regime. Leave-one-out-cross-validation to the whole procedure was not possible with the current study design given that the 9-gene classifier was derived in two stages: 1) from the intersection of three gene lists from three separate analyses, and 2) from only those genes confirmed by QRT-PCR. We acknowledge that overfitting could play a role in this study, and for this reason we used a stringent filtering approach as outlined in Figure 1. The highest prediction accuracy achieved using the 9-gene classifier for individual *BRCA1*, *BRCA2* and BRCAX samples (59%) was similar to that previously reported by Waddell et al (62%) [9], although due to differences in experimental design we cannot exclude the possibility that the prediction accuracy from the latter study may have been influenced by an experimentally induced bias. Importantly, our results showed that after treatment with MMC, *BRCA1* and *BRCA2* samples were shown here to be more dissimilar than either *BRCA1* or *BRCA2* was from BRCAX. Furthermore, in contrast to Waddell et al [9], *BRCA1* and *BRCA2* samples were classified with high accuracy, thus supporting the notion that LCLs harboring pathogenic mutations in *BRCA1* and *BRCA2* have a distinctive expression. Together these results suggest that compared to *BRCA1* and *BRCA2* truncating mutation carriers BRCAX comprises a genetically heterogeneous group that requires further molecular-based stratification. This notion is also consistent with linkage studies [16] as well as molecular studies that suggested BRCAX tumors can be classified into at least five molecular subtypes [17],[18]. It is therefore reasonable to propose that the accuracy of classifying pathogenic sequence variants in LCLs by expression profiling will improve as molecular subgroups within BRCAX individuals are identified.

An important method employed by this microarray-based study was the use of RNA pooling primarily to reduce cost. Studies have also used RNA pooling as a strategy to reduce the effects of biological variation with the aim of detecting gene expression profiles that differ between biological class [14],[15]. A disadvantage with pooling RNA is the impact it may have on statistical power in identifying genes that are differentially expressed between two or more classes [19],[20]. This is because pooling RNA prevents both accurate measurement of expression variation within the sample population and identification of deviant samples. Pooling has been shown to be most useful when the gene expression differences between biological conditions are larger than differences introduced by technical variability [21]–[23]. LCLs analyzed in the present study showed relatively low biological variation between pools for many of the genes analyzed, including the nine genes found to be 1.2 µM MMC(T_60_)-responsive. Expression differences between the biological classes studied were therefore more prone to variance introduced at each step of the microarray experiment. These small differences may account in part for the reduced classification accuracy observed using expression values measured by QRT-PCR as compared to the same analysis using microarray data. However, it is worth noting that we generally observed good correlation between the RNA pools and virtual pools for the expression differences (Table S2), supporting the use of pooling sample RNA for initial microarray experiments to direct downstream analysis of individual samples.

Previous studies have suggested MMC may perturb the Fanconi anemia pathway, in which BRCA2 plays a major role [24]. Interestingly, the protein encoded by one of the nine MMC responsive genes, CASP3, is known to be activated by the Fanconi anemia pathway as result of MMC or IR treatment [25]. The nuclear lamina protein LMNA has also been shown to play a role in ATR mediated DNA repair [26] and through this role may interact with BRCA1 and/or BRCA2 in response to MMC induced DNA damage [27]. It is unclear at this stage whether the remaining seven MMC responsive genes play a role in the Fanconi anemia pathway, and how they are functionally linked to BRCA1 and/or BRCA2. It is possible that unmapped BRCA1- and/or BRCA2-related pathways are also being perturbed by MMC treatment. An intriguing thought is the possibility that these genes may act as potential modifiers of *BRCA1* and/or *BRCA2* associated breast cancer risk. We have previously reported a novel method of using expression arrays and the Cancer Genetic Markers of Susceptibility (CGEMS) Breast Cancer Whole Genome Association Scan to prioritize IR response genes that potentially modify breast cancer risk in *BRCA1* and *BRCA2* carriers [28]. It is interesting to note that of the nine 1.2 µM MMC(T_60_)-responsive genes, *GPM6A* and *CDCP1* are tagged with single nucleotide polymorphisms that are shown by CGEMS to be associated with breast cancer risk (P<0.05) (data not shown). Furthermore, deletions of chromosome regions harboring *GPM6* (4q34.2), *CASP3* (4q35.1), and *P4HA2* (5q23.3) have been shown to be associated with breast tumors from *BRCA1* mutation carriers [29]–[31],[18]. Likewise, genomic regions harboring *RARSL* (6q15) and *FAM26F* (6q22.1) have been frequently deleted in *BRCA2* associated breast tumors [31]. These results give rise to an intriguing possibility that *GPM6*, *CASP3*, *P4HA2, RARSL* and *FAM26F* may also be targeted during breast tumorigenesis as the tumor cells undergo genomic copy number change.

In summary, our results demonstrate the use of RNA pooling and microarray profiling to assess LCLs derived from patients with a strong family history of breast cancer. This study highlights the novel use of MMC to perturb LCL expression profiles to identify genes that correlate with *BRCA1*, *BRCA2* and BRCAX mutation status. This strategy proved promising for classifying mutation status by gene expression profile, particularly between *BRCA1* and *BRCA2*, and prediction accuracy may be improved further by exploring different MMC doses and/or analysis time points. We propose that the pooling method is the most practical approach for comparing a number of different treatment regimes across several different sample sets. This strategy is likely to be very useful for identifying treatments that induce the greatest expression changes in LCLs after stimulation. Identifying genes whose expression is associated with *BRCA1*, *BRCA2* and BRCAX mutation status would be a valuable method of screening individuals from multiple case breast cancer families for the presence of pathogenic mutations.

## Materials and Methods

### Ethics statement

Ethical approvals were obtained from the Human Research Ethics Committees of the Queensland Institute of Medical Research and the Peter MacCallum Cancer Centre. Written informed consent was obtained from each participant.

### Subjects and lymphoblastoid cell-lines

Epstein Barr virus-transformed lymphoblastoid cell-lines (LCLs) were derived from breast cancer-affected women in multi-case families recruited into the Kathleen Cuningham Foundation for Research into Breast Cancer (kConFab) [32] and from healthy female controls recruited as volunteers from the Queensland Institute of Medical Research. A cohort of 36 LCLs were used in this study, including nine LCLs from women carrying a pathogenic mutation in *BRCA1*, nine LCLs from women carrying a pathogenic mutation in *BRCA2*, nine LCLs from women from breast cancer families that have tested negative for pathogenic mutations in *BRCA1* or *BRCA2* (termed BRCAX), and nine LCLs from healthy control females. Details of the mutations carried by each of the LCLs used in the study are shown in Table S12.

### LCL culture and treatment

LCLs were cultured in RPMI-1640 (Gibco Invitrogen) supplemented with 10% Serum Supreme (Lonza BioWhittaker), 1% penicillin-streptomycin (Gibco Invitrogen). Cell number was normalized to a density of 5×10^5^ cells/mL, approximately 4 h prior to treatment. To extend a previous study where gene expression levels were measured in LCLs after 10 Gy IR and 30 minute incubation [9], this study aims to identify IR responsive genes after an equivalent IR dose but at 60 minutes post-treatment. The MMC treatments were selected based on previous reports that showed LCLs carrying a mutation in the *BRCA2* gene were sensitive to MMC at 0.05 µM - 1.2 µM after 1–2 hours incubation [33],[34]. In this study, LCLs from each of the *BRCA1*, *BRCA2* and BRCAX patient groups, and from healthy controls, were irradiated at 10 Gy using a calibrated Cesium-137 source or treated with MMC at two different doses (0.4 µM or 1.2 µM). Cells were harvested prior to IR or MMC treatment (T_0_), at 1 h after IR exposure, and at 1 and 2 h after exposure for MMC.

### Microarray expression profiling

Total RNA was extracted and purified using the RNeasy Mini Kit (Qiagen GmbH). Three RNA pools were generated within each group (*BRCA1, BRCA2*, BRCAX and healthy controls) that comprised RNA (1000 ng) from each of three individual samples. RNA was quantified pre- and post-pooling using the NanoDrop ND-1000 spectrophotometer (Thermo Scientific). A comparison of estimated and observed RNA concentrations associated with each pool is detailed in Table S13. This procedure was carried out for each of the six treatment groups (including T_0_), thus generating a total of 72 RNA pools. The Illumina TotalPrep RNA Amplification Kit (Ambion) was used to amplify and biotinylate 450 ng of total RNA from each of the pools. Biotinylated RNA was hybridized to Illumina HumanRef8-V2 Beadchips (∼22,000 probes), washed, and stained with streptavidin-Cy3 before scanning with an Illumina BeadArray Reader. The RNA pools were processed in random order to minimize any chance of technical bias being introduced into the microarray data. Duplicate arrays were performed for eight pools to test for reproducibility, and a high correlation (r^2^>0.99) was measured within each paired-pool comparison. Only one of each duplicated sample was included in subsequent analyses.

### Microarray data analysis

Raw data were processed using Illumina BeadStudio before undergoing quantile normalization to account for systematic variation between arrays. Microarray data are available via GEO: GSE17764. Probes that obtained an Illumina detection score greater than 0.99 in at least one of the arrays (n = 16,478 probes) were retained for further analysis. Subsequent statistical analysis of genes differentially expressed between RNA-pools, classified by mutation and treatment type, was carried out using BRB-ArrayTools version 3.7.0 (http://linus.nci.nih.gov/BRB-ArrayTools.html). Genes differentially expressed between *BRCA1*, *BRCA2* and BRCAX pools, and between treated and untreated LCL pairs of healthy control pools were evaluated using three-sample F-tests and paired T-tests, respectively (α = 0.05). Microarray expression profiles of the treated and untreated LCL pairs of *BRCA1*, *BRCA2* and BRCAX pools were compared using paired T-tests and the number of false discoveries was restricted to 10% at a 90% confidence level using methods described elsewhere [35],[36].

### Quantitative reverse transcription–PCR

First-strand cDNA synthesis was performed using 450 ng of total RNA and SuperScript III First-Strand Synthesis System for RT-PCR (Invitrogen), according to manufacturer's instructions. Quantitative reverse transcription PCR (QRT-PCR) was performed using Platinum SYBR Green qPCR SuperMix-UDG (Invitrogen) and the LightCycler480 system (Roche Applied Science). Briefly, each 15 µL reaction contained 1x Platinum SYBR Green qPCR SuperMix-UDG, and 333 nM of each primer. Primer sequences are listed in Table S14. For each gene, primers sequences were designed to target at least one exon detected by the Illumina HumanRef8-V2 Beadchip probe sequence. QRT-PCR conditions were as follows: 50°C for 2 minutes, 95°C for 2 minutes, and then 45 cycles of 95°C for 20 seconds, 60°C for 15 seconds and 72°C for 20 seconds. All QRT-PCR reactions were done in triplicate. The data were normalized to the housekeeping gene *EEF1A1* and log_2_-transformed for further analysis.

### Class prediction with microarray and QRT–PCR data

Class prediction was performed using Diagonal Linear Discriminant Analysis [37], K-Nearest Neighbour Classification [37], Nearest Centroid [38], Support Vector Machines (SVM) [39], and Compound Covariate Predictor [40] algorithms in BRB-ArrayTools version 3.7.0. The K-Nearest Neighbour method used one nearest neighbour (*k* = 1), and the linear kernel method was used for Support Vector Machines. The models incorporated MMC responsive genes confirmed by QRT-PCR (see Results) that were differentially expressed between *BRCA1*, *BRCA2* and BRCAX classes. Leave-one-out cross-validation method was used to compute misclassification rate [41].

## Supporting Information

Figure S1 Supervised cluster analysis of IR treated (IR T60) and non-treated (T0) RNA pools from *BRCA1* and *BRCA2* mutation carriers, non-*BRCA1/2* (BRCAX) carriers and healthy control (HC) individuals using 19 genes (*ATM, BRCA1, CDKN1A, CHEK1, CHEK2, GADD45A, JUN, MAPK8, MDM2, MRE11A, MTTP, NBN, NFKB1, NFKBIA, RAD50, RAD51, RBBP8, TP53, TP73*) comprising the ATM Signaling Pathway (Biocarta).
(0.11 MB TIF)

Table S1 List of genes and their associated significance levels from three different analyses.
(0.52 MB XLS)

Table S2 Correlation of QRT-PCR derived expression data between pools and virtual pools.
(0.05 MB DOC)

Table S3 Performance of classifier with *BRCA1*, *BRCA2*, and BRCAX pools during cross-validation.
(0.05 MB DOC)

Table S4 Predictions of classifiers for *BRCA1*, *BRCA2*, and BRCAX virtual pools and samples.
(0.06 MB DOC)

Table S5 Correct classification rates of *BRCA1*, *BRCA2*, and BRCAX pools using microarray data from the various treatment groups and the nine 1.2 µM MMC(T_60_)-responsive genes.
(0.03 MB DOC)

Table S6 Performance of classifier with *BRCA1* and *BRCA2* pools during cross-validation.
(0.05 MB DOC)

Table S7 Predictions of classifiers for *BRCA1* and *BRCA2* virtual pools and samples.
(0.06 MB DOC)

Table S8 Performance of classifier with *BRCA1* and BRCAX pools during cross-validation.
(0.05 MB DOC)

Table S9 Predictions of classifiers for *BRCA1* and BRCAX virtual pools and samples.
(0.06 MB DOC)

Table S10 Performance of classifier with *BRCA2* and BRCAX pools during cross-validation.
(0.05 MB DOC)

Table S11 Predictions of classifiers for *BRCA2* and BRCAX virtual pools and samples.
(0.06 MB DOC)

Table S12 Details of mutations carried by each LCL used in the study and pool assignment.
(0.05 MB DOC)

Table S13 Comparison of estimated and observed RNA concentrations associated with each pool analysed.
(0.04 MB DOC)

Table S14 QRT-PCR primer details.
(0.05 MB DOC)
