# Supplementary figures and images for: Use of DNA–Damaging Agents and RNA Pooling to Assess Expression Profiles Associated with BRCA1 and BRCA2 Mutation Status in Familial Breast Cancer Patients
Source: PLoS Genet. 2010 Feb 19;6(2):e1000850. doi: 10.1371/journal.pgen.1000850 (PMC2824809; doi:10.1371/journal.pgen.1000850)

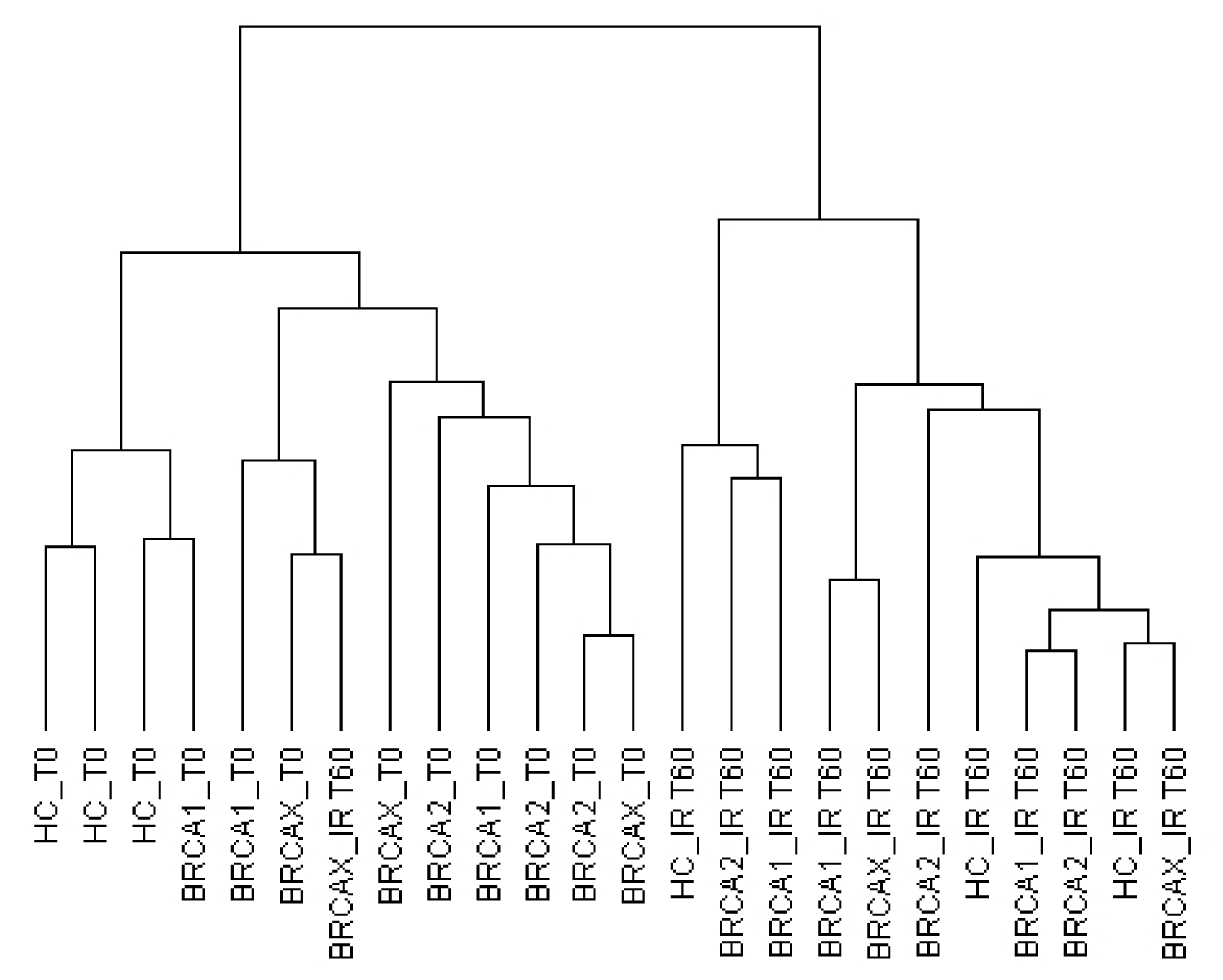

Supplement: Figure S1 — Supervised cluster analysis of IR treated (IR T60) and non-treated (T0) RNA pools from BRCA1 and BRCA2 mutation carriers, non-BRCA1/2 (BRCAX) carriers and healthy control (HC) individuals using 19 genes (ATM, BRCA1, CDKN1A, CHEK1, CHEK2, GADD45A, JUN, MAPK8, MDM2, MRE11A, MTTP, NBN, NFKB1, NFKBIA, RAD50, RAD51, RBBP8, TP53, TP73) comprising the ATM Signaling Pathway (Biocarta). (0.11 MB TIF) [file pgen.1000850.s001.tif]
