# Supplementary material for: Use of DNA–Damaging Agents and RNA Pooling to Assess Expression Profiles Associated with BRCA1 and BRCA2 Mutation Status in Familial Breast Cancer Patients
Source: PLoS Genet. 2010 Feb 19;6(2):e1000850. doi: 10.1371/journal.pgen.1000850 (PMC2824809; doi:10.1371/journal.pgen.1000850)
Supplement: Table S2 — Correlation of QRT-PCR derived expression data between pools and virtual pools. (0.05 MB DOC) [file pgen.1000850.s003.doc]

**Table S2**

Correlation of QRT-PCR derived expression data between pools and virtual pools.

|  | ***LMNA*** | ***RARSL*** | ***CDCP1*** | ***FAM26F*** | ***P4HA2*** | ***GPM6A*** | ***CCDC151*** | ***CASP3*** | ***SLC6A4*** | **r^a^** |
| --- | --- | --- | --- | --- | --- | --- | --- | --- | --- | --- |
| **BRCA1 Pool 1** | -7.39 | -11.66 | -14.92 | -11.63 | -8.08 | -17.29 | -13.27 | -7.50 | -10.94 | 0.99 |
| **BRCA1 Virtual Pool 1** | -8.01 | -11.87 | -15.08 | -12.26 | -8.85 | -17.79 | -13.49 | -7.85 | -10.23 |  |
| **BRCA1 Pool 2** | -7.83 | -12.27 | -14.18 | -10.70 | -8.23 | -15.99 | -12.52 | -7.94 | -10.43 | 0.99 |
| **BRCA1 Virtual Pool 2** | -8.49 | -12.04 | -14.35 | -11.03 | -8.87 | -16.27 | -12.21 | -7.53 | -9.67 |  |
| **BRCA1 Pool 3** | -7.52 | -11.81 | -13.52 | -11.19 | -8.04 | -15.46 | -13.31 | -7.26 | -11.09 | 0.99 |
| **BRCA1 Virtual Pool 3** | -8.10 | -12.55 | -13.97 | -11.95 | -8.25 | -16.44 | -13.18 | -7.63 | -11.00 |  |
| **BRCA2 Pool 1** | -7.53 | -11.02 | -14.60 | -10.52 | -8.50 | -14.87 | -12.14 | -7.99 | -11.16 | 0.99 |
| **BRCA2 Virtual Pool 1** | -8.24 | -11.21 | -15.35 | -10.79 | -8.67 | -15.16 | -12.03 | -8.04 | -10.96 |  |
| **BRCA2 Pool 2** | -7.76 | -10.81 | -13.83 | -10.06 | -8.70 | -13.80 | -12.12 | -7.96 | -11.76 | 0.93 |
| **BRCA2 Virtual Pool 2** | -8.79 | -11.25 | -13.45 | -9.50 | -8.88 | -15.83 | -11.20 | -8.09 | -11.41 |  |
| **BRCA2 Pool 3** | -8.48 | -10.84 | -13.29 | -10.77 | -8.36 | -15.44 | -11.91 | -7.80 | -10.43 | 0.97 |
| **BRCA2 Virtual Pool 3** | -9.48 | -10.84 | -14.28 | -11.11 | -8.46 | -14.81 | -11.81 | -7.80 | -9.92 |  |
| **BRCAX Pool 1** | -8.27 | -12.32 | -17.12 | -10.93 | -8.21 | -16.63 | -12.71 | -7.59 | -9.70 | 1.00 |
| **BRCAX Virtual Pool 1** | -8.79 | -12.64 | -16.81 | -11.29 | -8.76 | -16.77 | -13.21 | -8.14 | -9.90 |  |
| **BRCAX Pool 2** | -8.09 | -10.80 | -14.10 | -11.14 | -8.18 | -14.88 | -11.58 | -7.69 | -10.12 | 0.99 |
| **BRCAX Virtual Pool 2** | -8.62 | -11.58 | -14.67 | -12.28 | -8.58 | -15.44 | -11.79 | -8.40 | -10.43 |  |
| **BRCAX Pool 3** | -8.71 | -10.79 | -14.69 | -11.16 | -8.70 | -15.99 | -11.84 | -7.56 | -9.84 | 0.99 |
| **BRCAX Virtual Pool 3** | -8.82 | -11.72 | -15.17 | -11.84 | -8.50 | -17.39 | -12.12 | -8.35 | -10.33 |  |
| **r^a^** | 0.82 | 0.81 | 0.92 | 0.95 | 0.16 | 0.71 | 0.85 | 0.04 | 0.76 |  |

^a^ Pearson Correlation
