# Supplementary material for: Use of DNA–Damaging Agents and RNA Pooling to Assess Expression Profiles Associated with BRCA1 and BRCA2 Mutation Status in Familial Breast Cancer Patients
Source: PLoS Genet. 2010 Feb 19;6(2):e1000850. doi: 10.1371/journal.pgen.1000850 (PMC2824809; doi:10.1371/journal.pgen.1000850)
Supplement: Table S5 — Correct classification rates of BRCA1, BRCA2, and BRCAX pools using microarray data from the various treatment groups and the nine 1.2 µM MMC(T60)-responsive genes. (0.03 MB DOC) [file pgen.1000850.s006.doc]

**Table S5**

Correct classification rates of *BRCA1*, *BRCA2* and BRCAX pools using microarray data from the various treatment groups and the nine 1.2 μM MMC(T_60_)-responsive genes.

| **Treatment** | **Mean percent of correct classification** | | |
| --- | --- | --- | --- |
|  | DLDA | 1-NN | NC |
| T_0_ | 33% | 56% | 44% |
| 0.4 μM MMC(T_60_) | 89% | 78% | 78% |
| 1.2 μM MMC(T_60_) | 100% | 67% | 89% |
| 0.4 μM MMC(T_120_) | 67% | 78% | 78% |
| 1.2 μM MMC(T_120_) | 89% | 89% | 78% |
| IR(T_60_) | 44% | 67% | 78% |

Abbreviations: DLDA, Diagonal Linear Discriminant Analysis; NC, Nearest Centroid; 1-NN, Nearest Neighbour.
