# Supplementary material for: Use of DNA–Damaging Agents and RNA Pooling to Assess Expression Profiles Associated with BRCA1 and BRCA2 Mutation Status in Familial Breast Cancer Patients
Source: PLoS Genet. 2010 Feb 19;6(2):e1000850. doi: 10.1371/journal.pgen.1000850 (PMC2824809; doi:10.1371/journal.pgen.1000850)
Supplement: Table S6 — Performance of classifier with BRCA1 and BRCA2 pools during cross-validation. (0.05 MB DOC) [file pgen.1000850.s007.doc]

**Table S6**

Performance of classifier with *BRCA1* and *BRCA2* pools during cross-validation

| **Expression data source** | **Class** |  | **DLDA** | **1-NN** | **NC** | **SVM** | **CCP** |
| --- | --- | --- | --- | --- | --- | --- | --- |
| Pools (Microarray) | BRCA1 | Pool 1 | YES | YES | YES | YES | YES |
|  |  | Pool 2 | YES | YES | YES | YES | YES |
|  |  | Pool 3 | YES | YES | YES | YES | YES |
|  | BRCA2 | Pool 1 | YES | YES | YES | YES | YES |
|  |  | Pool 2 | NO | YES | YES | YES | YES |
|  |  | Pool 3 | YES | YES | YES | YES | YES |
|  | BRCA1 | Sensitivity | 1.00 | 1.00 | 1.00 | 1.00 | 1.00 |
|  |  | Specificity | 0.67 | 1.00 | 1.00 | 1.00 | 1.00 |
|  | BRCA2 | Sensitivity | 0.67 | 1.00 | 1.00 | 1.00 | 1.00 |
|  |  | Specificity | 1.00 | 1.00 | 1.00 | 1.00 | 1.00 |
| Pools (QRT-PCR) | BRCA1 | Pool 1 | YES | YES | YES | YES | YES |
|  |  | Pool 2 | YES | YES | YES | YES | YES |
|  |  | Pool 3 | YES | YES | YES | YES | YES |
|  | BRCA2 | Pool 1 | YES | YES | YES | YES | YES |
|  |  | Pool 2 | YES | YES | YES | YES | YES |
|  |  | Pool 3 | YES | YES | YES | NO | YES |
|  | BRCA1 | Sensitivity | 1.00 | 1.00 | 1.00 | 1.00 | 1.00 |
|  |  | Specificity | 1.00 | 1.00 | 1.00 | 0.67 | 1.00 |
|  | BRCA2 | Sensitivity | 1.00 | 1.00 | 1.00 | 0.67 | 1.00 |
|  |  | Specificity | 1.00 | 1.00 | 1.00 | 1.00 | 1.00 |
