# Supplementary material for: Use of DNA–Damaging Agents and RNA Pooling to Assess Expression Profiles Associated with BRCA1 and BRCA2 Mutation Status in Familial Breast Cancer Patients
Source: PLoS Genet. 2010 Feb 19;6(2):e1000850. doi: 10.1371/journal.pgen.1000850 (PMC2824809; doi:10.1371/journal.pgen.1000850)
Supplement: Table S9 — Predictions of classifiers for BRCA1 and BRCAX virtual pools and samples. (0.06 MB DOC) [file pgen.1000850.s010.doc]

**Table S9**

Predictions of classifiers for *BRCA1* and BRCAX virtual pools and samples

| **Expression data source** | **Class** |  | **DLDA** | **1-NN** | **NC** | **SVM** | **CCP** |
| --- | --- | --- | --- | --- | --- | --- | --- |
| Virtual Pools (QRT-PCR) | BRCA1 | Pool 1 | BRCA1 | BRCA1 | BRCA1 | BRCA1 | BRCA1 |
|  |  | Pool 2 | BRCAX | BRCA1 | BRCAX | BRCA1 | BRCAX |
|  |  | Pool 3 | BRCA1 | BRCA1 | BRCA1 | BRCA1 | BRCA1 |
|  | BRCAX | Pool 1 | BRCAX | BRCAX | BRCAX | BRCAX | BRCAX |
|  |  | Pool 2 | BRCAX | BRCAX | BRCAX | BRCAX | BRCAX |
|  |  | Pool 3 | BRCAX | BRCAX | BRCAX | BRCAX | BRCAX |
|  | BRCA1 | Sensitivity | 0.67 | 1.00 | 0.67 | 1.00 | 0.67 |
|  |  | Specificity | 1.00 | 1.00 | 1.00 | 1.00 | 1.00 |
|  | BRCAX | Sensitivity | 1.00 | 1.00 | 1.00 | 1.00 | 1.00 |
|  |  | Specificity | 0.67 | 1.00 | 0.67 | 1.00 | 0.67 |
| Individuals (QRT-PCR) | BRCA1 | Sample 1 | BRCAX | BRCA1 | BRCA1 | BRCAX | BRCAX |
|  |  | Sample 2 | BRCAX | BRCAX | BRCAX | BRCAX | BRCAX |
|  |  | Sample 3 | BRCA1 | BRCA1 | BRCA1 | BRCA1 | BRCA1 |
|  |  | Sample 4 | BRCA1 | BRCA1 | BRCA1 | BRCA1 | BRCA1 |
|  |  | Sample 5 | BRCAX | BRCAX | BRCA1 | BRCAX | BRCAX |
|  |  | Sample 6 | BRCAX | BRCAX | BRCAX | BRCAX | BRCAX |
|  |  | Sample 7 | BRCA1 | BRCA1 | BRCA1 | BRCA1 | BRCA1 |
|  |  | Sample 8 | BRCA1 | BRCA1 | BRCA1 | BRCA1 | BRCA1 |
|  |  | Sample 9 | BRCA1 | BRCA1 | BRCA1 | BRCA1 | BRCA1 |
|  | BRCAX | Sample 19 | BRCAX | BRCAX | BRCAX | BRCAX | BRCAX |
|  |  | Sample 20 | BRCAX | BRCAX | BRCAX | BRCAX | BRCAX |
|  |  | Sample 21 | BRCA1 | BRCA1 | BRCA1 | BRCA1 | BRCA1 |
|  |  | Sample 22 | BRCA1 | BRCAX | BRCAX | BRCAX | BRCA1 |
|  |  | Sample 23 | BRCAX | BRCA1 | BRCAX | BRCA1 | BRCAX |
|  |  | Sample 24 | BRCAX | BRCAX | BRCAX | BRCAX | BRCAX |
|  |  | Sample 25 | BRCA1 | BRCA1 | BRCA1 | BRCA1 | BRCA1 |
|  |  | Sample 26 | BRCAX | BRCA1 | BRCA1 | BRCAX | BRCAX |
|  |  | Sample 27 | BRCA1 | BRCA1 | BRCA1 | BRCA1 | BRCA1 |
|  | BRCA1 | Sensitivity | 0.56 | 0.67 | 0.78 | 0.56 | 0.56 |
|  |  | Specificity | 0.56 | 0.44 | 0.56 | 0.56 | 0.56 |
|  | BRCAX | Sensitivity | 0.56 | 0.44 | 0.56 | 0.56 | 0.56 |
|  |  | Specificity | 0.67 | 0.67 | 1.00 | 0.67 | 0.67 |
