# Supplementary material for: Use of DNA–Damaging Agents and RNA Pooling to Assess Expression Profiles Associated with BRCA1 and BRCA2 Mutation Status in Familial Breast Cancer Patients
Source: PLoS Genet. 2010 Feb 19;6(2):e1000850. doi: 10.1371/journal.pgen.1000850 (PMC2824809; doi:10.1371/journal.pgen.1000850)
Supplement: Table S12 — Details of mutations carried by each LCL used in the study and pool assignment. (0.05 MB DOC) [file pgen.1000850.s013.doc]

**Table S12**

Details of mutations carried by each LCL used in the study and pool assignment.

| **SAMPLE NUMBER** | **MUTATION CLASS** | **MUTATION** | **Pool assignment** |
| --- | --- | --- | --- |
| 1 | *BRCA1* | *BRCA1* 2800_2801 del AA (STOP 901) | 1 |
| 2 | *BRCA1* | *BRCA1* 2829 G>T (E904X) | 1 |
| 3 | *BRCA1* | *BRCA1* 2138 del A (STOP 700) | 1 |
| 4 | *BRCA1* | *BRCA1* 3450_3453 del CAAG (STOP 1115) | 2 |
| 5 | *BRCA1* | *BRCA1* del exon 21_24 (c5397-?_5711+?del) | 2 |
| 6 | *BRCA1* | *BRCA1* 184_185 ins TT (STOP 23) | 2 |
| 7 | *BRCA1* | *BRCA1* 2681_2682 ins GC (STOP 893) | 3 |
| 8 | *BRCA1* | *BRCA1* 1590 C>T exon 11 (Q491X) | 3 |
| 9 | *BRCA1* | *BRCA1* 1406_1407 ins A (STOP 434) | 3 |
| 10 | *BRCA2* | *BRCA2* del exons 1_2 (g54799-?_55847+?del) | 1 |
| 11 | *BRCA2* | *BRCA2* 1617_1618 del AG (STOP 466) | 1 |
| 12 | *BRCA2* | *BRCA2* 6503_6504 del TT (STOP 2098) | 1 |
| 13 | *BRCA2* | *BRCA2* 7985 G>A (W2586X) | 2 |
| 14 | *BRCA2* | *BRCA2* 2000_2003 del TTTA (STOP 612) | 2 |
| 15 | *BRCA2* | *BRCA2* 4365_4369 del GATTA (STOP 1400)* | 2 |
| 16 | *BRCA2* | *BRCA2* 5572 C>T (Q1782X) | 3 |
| 17 | *BRCA2* | *BRCA2* 6024_6025 del TA (STOP 1943) | 3 |
| 18 | *BRCA2* | *BRCA2* 5910 C>G (Y1894X) | 3 |
| 19 | BRCAX | No mutation identified** | 1 |
| 20 | BRCAX | No mutation identified** | 1 |
| 21 | BRCAX | No mutation identified** | 1 |
| 22 | BRCAX | No mutation identified** | 2 |
| 23 | BRCAX | No mutation identified** | 2 |
| 24 | BRCAX | No mutation identified** | 2 |
| 25 | BRCAX | No mutation identified** | 3 |
| 26 | BRCAX | No mutation identified** | 3 |
| 27 | BRCAX | No mutation identified** | 3 |
| 28 | Healthy Control | No known mutation | 1 |
| 29 | Healthy Control | No known mutation | 1 |
| 30 | Healthy Control | No known mutation | 1 |
| 31 | Healthy Control | No known mutation | 2 |
| 32 | Healthy Control | No known mutation | 2 |
| 33 | Healthy Control | No known mutation | 2 |
| 34 | Healthy Control | No known mutation | 3 |
| 35 | Healthy Control | No known mutation | 3 |
| 36 | Healthy Control | No known mutation | 3 |

* also carries a *BRCA2* unclassified variant, *BRCA2* 6328 C>T (R2034C).

** Negative for pathogenic mutations in *BRCA1* and *BRCA2* after complete sequencing, and multiplex-ligation-dependent probe amplification gene dosage assay (MLPA) for detection of large genmoic rearrangements of *BRCA1* and *BRCA2.*
