# Supplementary material for: Use of DNA–Damaging Agents and RNA Pooling to Assess Expression Profiles Associated with BRCA1 and BRCA2 Mutation Status in Familial Breast Cancer Patients
Source: PLoS Genet. 2010 Feb 19;6(2):e1000850. doi: 10.1371/journal.pgen.1000850 (PMC2824809; doi:10.1371/journal.pgen.1000850)
Supplement: Table S13 — Comparison of estimated and observed RNA concentrations associated with each pool analysed. (0.04 MB DOC) [file pgen.1000850.s014.doc]

**Table S13**

Comparison of estimated^a^ and observed^b^ RNA concentrations associated with each pool analysed

| **Mutation class** | **Pool assignment** | **T_0_** | **IR (10 Gy)** | **MMC T_60_ 0.4µM** | **MMC T_120_ 0.4 µM** | **MMC T_60_ 1.2 µM** | **MMC T_120_ 1.2 µM** |
| --- | --- | --- | --- | --- | --- | --- | --- |
| *BRCA1* | Pool A1 | 419/393^c^ (7%)^d^ | 461/435 (6%) | 435/399 (9%) | 470/443 (6%) | 284/294 (3%) | 319/315 (1%) |
| *BRCA1* | Pool A2 | 418/398 (5%) | 678/651 (4%) | 678/649 (4%) | 707/681 (4%) | 610/598 (2%) | 432/399 (8%) |
| *BRCA1* | Pool A3 | 601/615 (2%) | 356/349 (2%) | 300/307 (2%) | 499/453 (10%) | 450/387 (16%) | 385/380 (1%) |
| *BRCA2* | Pool A1 | 542/529 (2%) | 385/369 (4%) | 310/296 (5%) | 514/521 (1%) | 414/393 (5%) | 578/565 (2%) |
| *BRCA2* | Pool A2 | 522/472 (11%) | 408/398 (2%) | 480/424 (13%) | 401/385 (4%) | 419/430 (3%) | 438/413 (6%) |
| *BRCA2* | Pool A3 | 476/442 (8%) | 527/472 (12%) | 432/411 (5%) | 398/373 (7%) | 342/332 (3%) | 347/336 (3%) |
| BRCAX | Pool A1 | 345/333 (4%) | 457/435 (5%) | 588/577 (2%) | 447/409 (9%) | 525/454 (16%) | 333/297 (12%) |
| BRCAX | Pool A2 | 460/433 (6%) | 401/374 (7%) | 404/387 (4%) | 486/443 (10%) | 448/422 (6%) | 406/378 (7%) |
| BRCAX | Pool A3 | 794/756 (5%) | 475/431 (10%) | 528/510 (3%) | 429/406 (6%) | 457/443 (3%) | 388/379 (2%) |
| Healthy Control | Pool A1 | 685/693 (1%) | 574/559 (3%) | 619/564 (10%) | 541/559 (3%) | 609/586 (4%) | 452/434 (4%) |
| Healthy Control | Pool A2 | 735/733 (0%) | 632/619 (2%) | 600/564 (6%) | 494/471 (5%) | 528/503 (5%) | 636/639 (0%) |
| Healthy Control | Pool A3 | 521/540 (3%) | 458/469 (2%) | 507/470 (8%) | 556/607 (8%) | 496/564 (12%) | 431/418 (3%) |

Abbreviations: IR, irradiation; MMC, mitomycin C

^a^ 3000(ng)/Final volume (ul) of pooled RNA

^b^ Concentration of pooled RNA measured using a Nanodrop (ng/ul)

^c^ [RNA_estimated_]/[RNA_observed_]

^d^ [RNA_estimated_]/[RNA_observed_]x100
