# Supplementary material for: Use of DNA–Damaging Agents and RNA Pooling to Assess Expression Profiles Associated with BRCA1 and BRCA2 Mutation Status in Familial Breast Cancer Patients
Source: PLoS Genet. 2010 Feb 19;6(2):e1000850. doi: 10.1371/journal.pgen.1000850 (PMC2824809; doi:10.1371/journal.pgen.1000850)
Supplement: Table S14 — QRT-PCR primer details. (0.05 MB DOC) [file pgen.1000850.s015.doc]

**Table S14**

QRT-PCR primer details

| **GB acc** | **Gene symbol** | **Forward primer sequence** | **Reverse primer sequence** |
| --- | --- | --- | --- |
| NM_016395 | PTPLAD1 | TCAACCTGACTGTGCGATTC | GCCAGCATCTGGCAGAAATA |
| NM_144636 | CHCHD4 | AGAATTGGTGGCTGATGACC | GCATGGGCAGTTCCAGTTA |
| NM_014056 | HIGD1A | CCCAAGGCTTTGTTGTAGGA | CCAACAAGACCAAGACAGCA |
| NM_021219 | JAM2 | AGGTGTCCTGGGAAACGAAT | CGGAAATCACTAAGGCCACA |
| NM_172200 | IL15RA | ACTGGAGGTCCCAGAGCAC | CAGATGTCTGCGTGTTCCAC |
| NM_005572 | LMNA | CCTGCGTACGGCTCTCAT | CGAGCGCAGGTTGTACTCA |
| NM_020320 | RARS2 | GTTCAGCTGGGATCGTGTTT | CATTCAGGTACCCACATCCA |
| NM_021170 | HES4 | ATCCTGGAGATGACCGTGAG | CGGTACTTGCCCAGAACG |
| NM_022842 | CDCP1 | GGCTCATCATTTGCTGTGTG | CTGCCTCGGCATCTCAGTAT |
| NM_144781 | PDCD2 | CCTGAGGTTGTGGAAAAGGA | TCCCTGGATTCATGTTTTGC |
| NM_001010919 | FAM26F | AGGATCTGAAGGCTCAGTCG | AGATAGGCATCGGGTGACAG |
| NM_052945 | TNFRSF13C | CCTGGTGGGTCTGGTGAG | ACCTTGTCCAGGGGCTCT |
| NM_004047 | ATP6V0B | GTCATCGTCGCAATTCTTCA | CCCAGGAAAACCAGCAATAA |
| NM_032383.3 | HPS3 | CCATGGGTTCACGTCGTAAT | TGGAAGCTATGTCAAATGAAGG |
| NM_001017974 | P4HA2 | GGGAAGGTGACTACCGAACA | ACTCCTGTCCTCGTTCATGG |
| NM_007043 | KRR1 | GACAAGAGGAAAGAAACAAAGCA | TGCTGGCCACATCAATTTTA |
| NM_003831 | RIOK3 | TCAGGCTTAAACATCACAGCA | TGAAGCAGCTTTCCTTCCAT |
| NM_014552 | GRHL1 | TGATGAGCATCAATGGAGATG | GGCTTTGCTGTTGATGACCT |
| NM_012229 | NT5C2 | TGCAGCATCTTTCATCAACC | GTGCTCCACCGTTGATTCAT |
| NM_021626 | SCPEP1 | TGGATTCTGAAAGCTGGTCAT | GCCCCATCCATCCTATTCTT |
| NM_005500 | SAE1 | TTTGGCACAGGAAATTGTGA | ACAATCCCATTCCCCTTCAT |
| NM_016410 | CHMP5 | GCACTGAGTCGCAGTTATGG | CATCAGCCAGAAGCTCATCA |
| NM_004147 | DRG1 | ACCGCTGGAATTTTGATGAC | GGTAACTGGCCTTTGGGTTT |
| NM_201592 | GPM6A | TATTGTGGCACTTGCTGGAG | GGCAGGCGTCTTTCACATAG |
| NM_005527 | HSPA1L | TTTCCAAACTGAAGCGAAGG | CCCACACAGGAGTAGGTGGT |
| NM_002147 | HOXB5 | TGAGGAAGCTTCACATCAGC | GAACTCCTTTTCCAGCTCCA |
| NM_144666 | DNHD1 | AACAGCAACCCTCTGCACTT | ACTCCGCATGTAGGACCTGT |
| NM_145045 | CCDC151 | TGCCACTTCCAAGGACAAGT | TTCTGGGAACGGATCTTGAG |
| NM_005516 | HLA-E | GGGGTCTGAGTCTCACAGCTT | CGTGAGGAAATCCTGCATCT |
| NM_004030 | IRF7 | GAGAAGAGCCTGGTCCTGGT | TGCTGCTATCCAGGGAAGAC |
| NM_005949 | MT1F | GCAAGTGCAAAGAGTGCAAA | GCCCCTTTGCAAACACAG |
| NM_203488 | ACYP1 | GCTGAAACTCACAGCAAGAGC | AGTGTTCTGGACCCAGCCTA |
| NM_032991 | CASP3 | GTGGAGGCCGACTTCTTGTA | ACAAAGCGACTGGATGAACC |
| NM_016564 | CEND1 | GAAACTCCCGAGCTCTCCTC | CCCCTGGGCATTCTATGG |
| NM_001045 | SLC6A4 | TTCATTTGCATCCCCACATA | CTGTTGGTGTTTCTGGGGTAA |
| NM_145755 | TTC21A | CAAGTTCGACCTCGCCTTAG | TCCATGATGAAGCCCATGTA |
| NM_001402 | EEF1A1 | AAAATGACCCACCAATGGAA | CGTGTGGCAATCCAATACAG |
